# A protocol for assessing virtual and mixed reality environments with real-time electromyographic (EMG) biofeedback during upper limb physical exercises

**DOI:** 10.1016/j.mex.2026.104122

**Published:** 2026-08-29

**Authors:** Mazen Talaat Haggag, Mohammed Ibrahim Awad, Ahmed Mohamed Elbokl, Bassem Amin Abdullah

**Affiliations:** a Department of Mechatronics Engineering, Faculty of Engineering, Ain Shams University, Cairo, Egypt; b Human Centered Mechatronics (HCM) Lab, Virtual Hospital, Ain Shams University, Cairo, Egypt; c Department of Neurology and Psychiatry, Faculty of Medicine, Ain Shams University, Cairo, Egypt; d Department of Computer and Systems Engineering, Faculty of Engineering, Ain Shams University, Cairo, Egypt

**Keywords:** Rehabilitation engineering, Shoulder exercise, Electromyography, Biofeedback, Kinematics, Motion capture

## Abstract

This paper describes a protocol for a controlled laboratory study designed to evaluate how immersive real-time electromyographic (EMG) biofeedback affects standardized shoulder exercise performance and user experience in healthy adults. Participants aged 20–30 years will be randomly allocated to virtual reality (VR), mixed reality (MR), or a non-immersive control condition. Within the immersive arms, each participant will experience three EMG visualization modes (an anatomically mapped avatar, a dynamic bar display, and a live waveform graph) in randomized order. During five standardized shoulder exercises, bilateral deltoid activity will be recorded using six wireless EMG sensors, while three-dimensional kinematics will be captured with an optical motion-capture system. Outcomes will include joint-angle accuracy, normalized muscle activation, compensatory motion, range of motion, movement speed, task completion time, together with perceived exertion, simulator sickness, and system usability. By integrating synchronized EMG and motion-capture data with immersive feedback delivery, this protocol provides a reproducible framework for comparing VR and MR biofeedback environments and evaluating how visualization mode may influence exercise execution and user experience.

• Compares VR, MR, and non-immersive shoulder exercise conditions.
• Evaluates avatar, bar, and waveform EMG visualization modes.
• Combines EMG, motion capture, and subjective assessments.

## Graphical abstract

**Figure:**
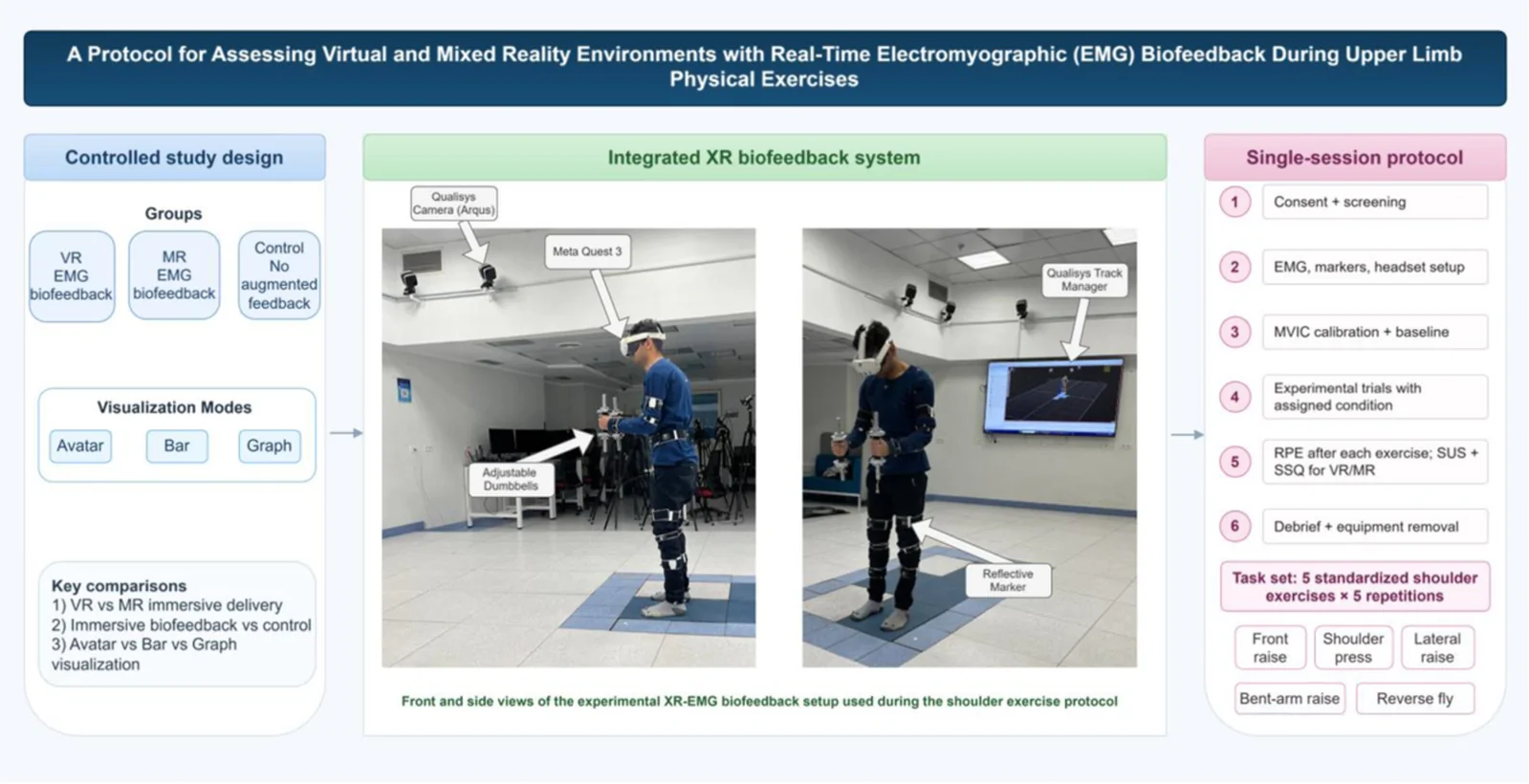

Specifications table

**Table:** 

**Subject area**	Engineering
**More specific subject area**	Rehabilitation engineering; immersive biofeedback; electromyography-based exercise assessment
**Name of your protocol**	A Protocol for Assessing Virtual and Mixed Reality Environments with Real-Time Electromyographic (EMG) Biofeedback During Upper Limb Physical Exercises
**Reagents/tools**	Meta Quest 3 HMD (Meta Platforms, Inc.); Delsys Trigno Avanti wireless EMG sensors (SP-W06) with Trigno Research+ Base Station (SP-W02); Qualisys motion capture system (12 Arqus A5 + 2 Miqus video cameras); Adjustable dumbbells (2–5 kg); Resistance bands; Workstation (Intel i9–11900K, NVIDIA RTX 3060 Ti 8 GB GPU, 32 GB DDR4 RAM, Windows 11); Unity Engine 2022.3.18f1 LTS; Qualisys Track Manager 2024.3; Qualisys Unity SDK Package 12; Python 3.11
**Experimental design**	Randomized controlled mixed laboratory protocol. Participants will be allocated to one of three between-subjects groups: virtual reality, mixed reality, or non-immersive control. Participants in the virtual and mixed reality groups will complete the exercise protocol using all three EMG visualization modes (avatar, bar, and graph) in randomized order. Control participants will complete the same exercises without augmented feedback. Each participant will complete a single ∼120 min session involving five standardized shoulder exercises. The target population is healthy adults aged 20–30 years.
**Trial registration**	None
**Ethics**	This protocol involves human participants; informed consent will be obtained from all participants prior to data collection. Ethical approval was obtained from the Institutional Review Board (IRB) of the Faculty of Engineering, Ain Shams University, Egypt (IRB code: IRB-ASU-25-017). The study protocol, informed consent documents, and all participant-facing materials were reviewed and approved by the IRB.
**Value of the Protocol**	• Provides a reproducible framework for integrating real-time EMG biofeedback with immersive virtual and mixed reality environments during shoulder exercise assessment. • Enables systematic comparison of three visualization strategies (avatar, bar, graph) for presenting physiological biofeedback in immersive settings. • Combines synchronized multimodal data (EMG + motion capture) with standardized subjective assessments in a single controlled protocol.

## Background

Shoulder rehabilitation and exercise-based shoulder assessment depend on accurate exercise performance to restore strength, range of motion, and function, yet correct technique is often difficult to maintain outside supervised settings. This challenge is especially relevant for shoulder disorders because reduced shoulder motion and strength can limit daily activities, while home-based rehabilitation technologies still require reliable ways to guide exercise quality and progression [[1], [2], [3]]. Conventional rehabilitation commonly relies on verbal instruction, intermittent observation, or motion-based feedback, which may provide limited real-time information about underlying muscle activation. As a result, individuals may complete the visible movement while using compensatory strategies, recruiting non-target muscles, or repeating suboptimal mechanics.

EMG biofeedback offers a way to make muscle activity visible during movement. In shoulder rehabilitation, systematic review evidence suggests that EMG biofeedback has been investigated for pain and function, but findings remain limited by small numbers of studies, heterogeneous protocols, and variable outcome measures [4]. Broader reviews of EMG combined with virtual or augmented reality also indicate that surface EMG can support rehabilitation applications as control or biofeedback, although standardized protocols remain limited [5]. Early systems combining EMG biofeedback with VR have focused mainly on upper-limb rehabilitation, usability, and task engagement rather than direct comparison of immersive delivery modes [6].

VR and MR offer more integrated ways to deliver feedback within the exercise experience. VR provides a fully computer-generated environment, whereas MR overlays digital content onto the physical environment, preserving visual access to the user’s body, equipment, and surroundings [7]. Both approaches are relevant to upper-limb rehabilitation because immersive environments can provide repetitive practice, salient feedback, task-oriented interaction, and motor-learning support [8]. Physiological measurements in VR may also support more objective and adaptive user-state assessment during immersive interaction [9].

A related unresolved issue is how EMG feedback should be visualized. Feedback design can influence attention, cognitive load, motor learning, and dependence on guidance [[10], [11], [12]]. Recent work on muscle-engagement visualization [13] and EMG-based interaction in VR further suggests that the body location, visual representation, and feedback modality can affect performance and workload [14]. However, the comparative effects of anatomically mapped avatar displays, abstract bar indicators, and live waveform graphs during shoulder exercises remain insufficiently understood.

Two methodological gaps, therefore, remain. First, direct comparisons between VR and MR for delivering real-time EMG biofeedback during shoulder exercises are limited. Second, few controlled protocols compare how different EMG visualization modes affect exercise execution and user experience. This protocol addresses these gaps by evaluating real-time EMG biofeedback during standardized shoulder exercises across VR, MR, and non-immersive conditions, while also comparing avatar-, bar-, and graph-based visualization modes. By combining objective kinematic and EMG outcomes with usability, exertion, and simulator-sickness measures, the study provides a structured workflow for quantifying exercise performance and user experience under controlled immersive biofeedback conditions.

## Description of protocol

### Study design

This study will use a controlled mixed experimental design. Participants will first be allocated to one of three between-subjects groups: (1) VR with real-time EMG biofeedback, (2) MR with real-time EMG biofeedback, or (3) a non-immersive control group that performs the same exercises without augmented feedback. This three-arm structure enables comparison between the two immersive delivery modes (VR vs. MR) and comparison between immersive biofeedback and conventional non-immersive exercise (VR and MR vs. control).

Within the VR and MR groups, each participant will experience all three EMG visualization modes: (1) an animated avatar with muscle-region highlighting, (2) a dynamic bar indicator, and (3) a live EMG waveform graph. The order of these visualization modes will be randomized for each participant to reduce order, learning, and fatigue effects. This within-participant visualization structure allows comparison of the three feedback formats while keeping the immersive delivery mode fixed for each participant. Participants in the control group will not receive visualization-format exposure because they perform the exercises without augmented feedback.

Randomization will be performed using a pre-prepared randomized allocation schedule designed to maintain balanced group distribution during recruitment. Participants will be assigned to the VR, MR, or non-immersive control group after eligibility confirmation and informed consent using a restricted randomization schedule with equal numbers of assignments arranged in randomized order. This ensures that participants are distributed evenly across the three study groups as recruitment progresses. For participants allocated to the VR and MR groups, the order of the EMG visualization modes will be randomized using a rotating visualization-order schedule. The avatar, bar, and graph modes will be assigned in different orders across participants so that the presentation sequence is balanced and order effects are reduced. Control participants will complete matched exercise blocks without EMG visualization. Group allocation and visualization-order records will be documented in the session log and stored separately from participant outcome files to maintain traceability.

### Setting

The study will be conducted at the Human Centered Mechatronics (HCM) Lab, located within the Virtual Hospital at Ain Shams University, Cairo, Egypt.

### Participants

#### Inclusion criteria

Participants will be eligible for inclusion if they meet all of the following criteria:

• Healthy adults aged 20 to 30 years
• No known history of shoulder injuries, pain, or movement disorders
• Normal or corrected-to-normal vision (to ensure adequate visibility of VR and MR displays)
• Ability to perform shoulder exercises at or above shoulder height without pain or restriction
• Not currently engaged in regular, intensive shoulder-specific training or rehabilitation programs

#### Exclusion criteria

Participants will be excluded if they meet any of the following criteria:

• Any diagnosed upper limb musculoskeletal or neurological impairment, or any uncorrected visual impairment (i.e., not correctable by standard glasses or contact lenses)
• History of motion sickness or significant discomfort when using VR or MR headsets
• Inability or unwillingness to wear EMG sensors or motion-capture markers
• Any other medical or physical condition that, in the judgment of the research team, could pose a risk or confound results

### Equipment and instruments

**Fig. 1:**
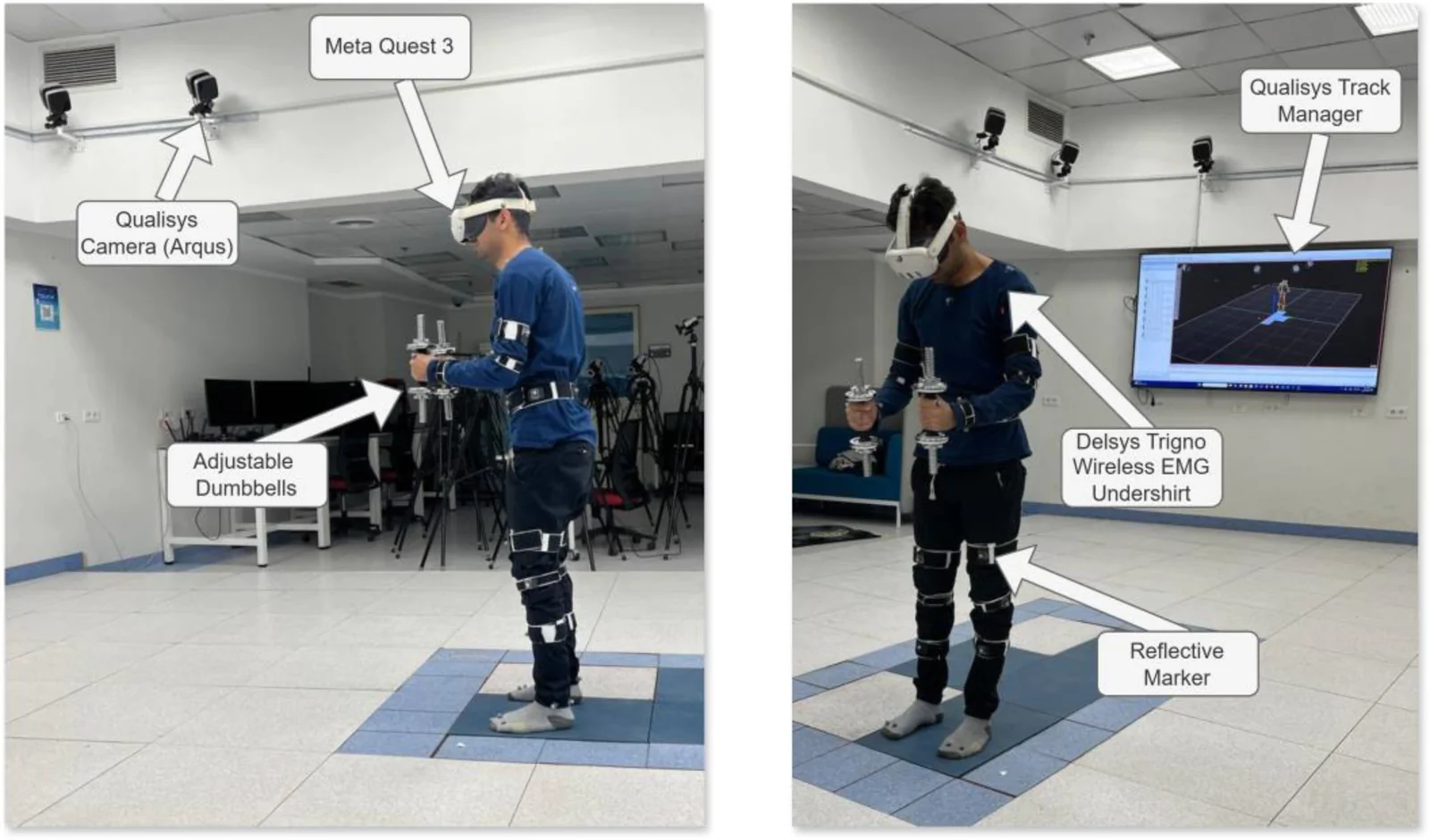
Experimental setup overview for the upper-limb XR biofeedback protocol. Participant wearing the Meta Quest 3 headset, wireless EMG sensors, and reflective markers while holding adjustable dumbbells within the Qualisys motion-capture volume with real-time motion tracking in Qualisys Track Manager (QTM).

The experimental setup will include an immersive display, motion-capture, EMG sensing, exercise, and computing components, as shown in Fig. 1.

• **Immersive display:** The Meta Quest 3 headset (Meta Platforms, Inc., Menlo Park, CA, USA) will be used for both VR and MR conditions. It provides standalone operation, inside-out tracking, high-resolution display, and full-color passthrough for MR feedback.
• **Motion capture:** Kinematic data will be captured using a Qualisys motion-capture system with 12 Arqus A5 and 2 Miqus video cameras arranged around an approximately 10.0 × 6.0 × 2.5 m capture volume. Cameras will be positioned approximately 2.9 m above the floor and angled inward/downward to maintain shoulder-marker visibility. Calibration will use an L-frame and T-wand, with a target residual error below 0.5 mm. Cameras will be synchronized at 300 Hz. Reflective markers will follow the Qualisys Sports Marker Set to compute joint angles, range of motion, velocity, and smoothness metrics.
• **EMG:** EMG data will be collected using six Delsys Trigno Avanti wireless sensors (Delsys Inc., Natick, MA, USA; Model SP-W06) connected to a Trigno Research+ Base Station (Model SP-W02). EMG will be sampled at 1926 Hz using the 20–450 Hz bandwidth setting. EMG and motion-capture recordings will be synchronized using a hardware TTL synchronization signal generated by the Qualisys Camera Sync Unit.
• **Exercise equipment:** Adjustable dumbbells (2–5 kg) and resistance bands of varying resistance levels will be used for exercise tasks and maximum voluntary isometric contraction (MVIC) calibration.
• **Computing infrastructure:** Data acquisition, VR and MR application development, and real-time signal processing will run on a workstation with an Intel i9–11900K processor, NVIDIA RTX 3060 Ti 8 GB GPU, 32 GB DDR4 RAM, and Windows 11. All data will be stored on secure, password-protected systems within the HCM Laboratory.

### System architecture and integration

**Fig. 2:**
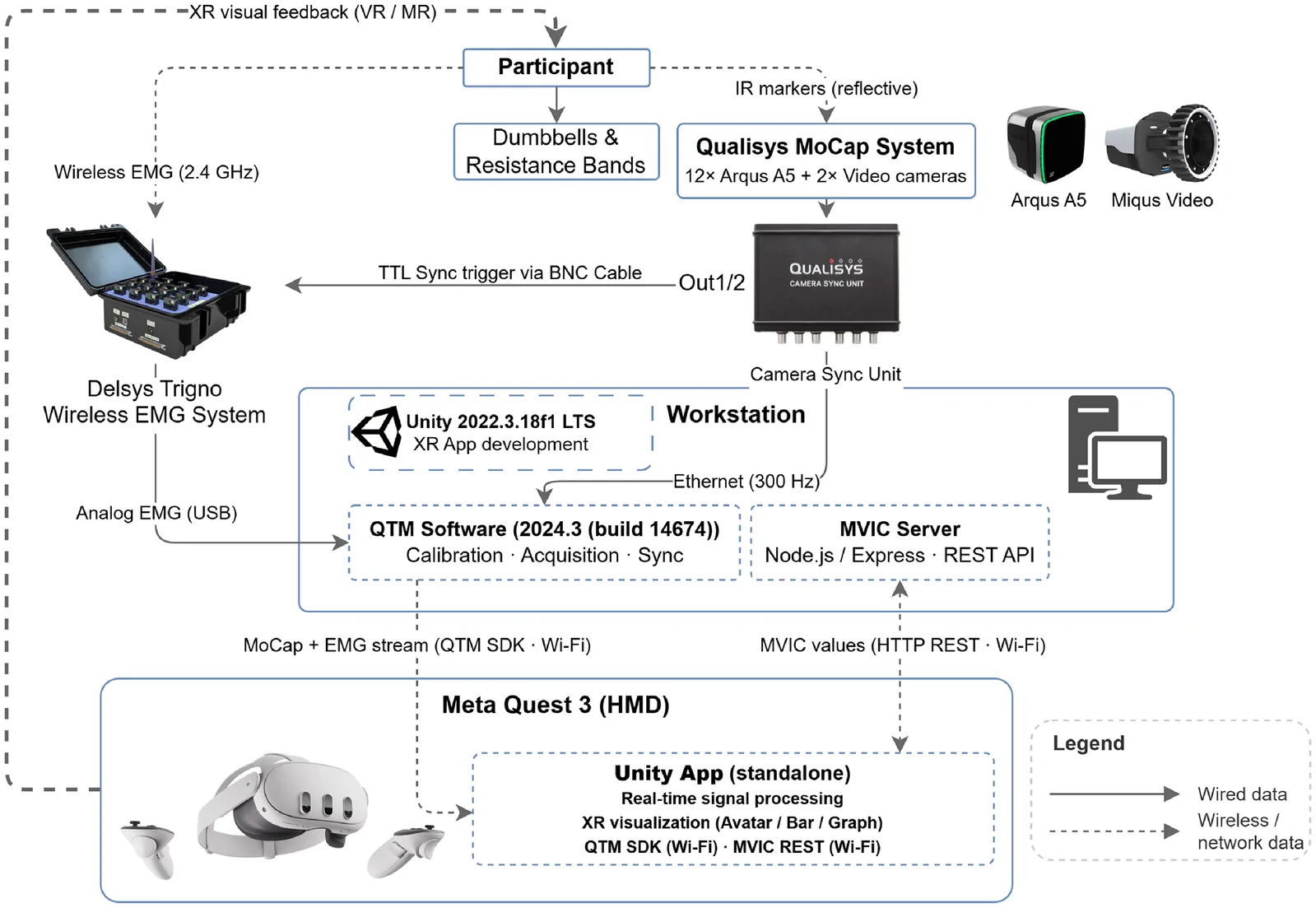
System architecture and integration.

The experimental system comprises four hardware subsystems and two software services operating as a single integrated pipeline. Fig. 2 illustrates the complete architecture; each component is described below.

**Fig. 3:**
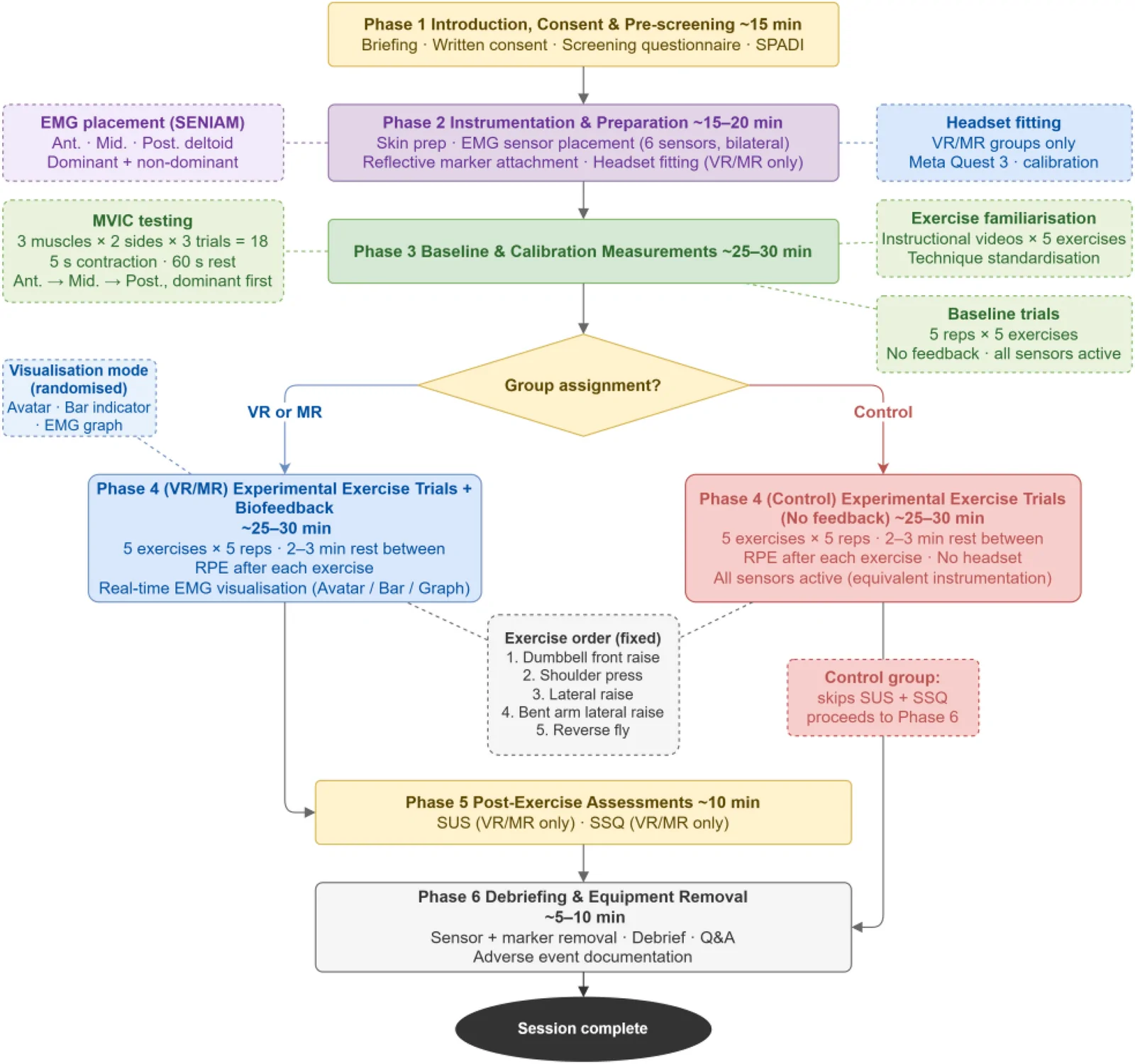
Deltoid EMG sensor placement regions illustrated on the right shoulder. The same placement procedure will be mirrored on the left shoulder.

**Participant.** The participant wears six Delsys Trigno Avanti wireless EMG sensors on the bilateral deltoid muscles (detailed in Fig. 3) and reflective markers on anatomical landmarks (detailed in Table 2). Exercises are performed using adjustable dumbbells or resistance bands, depending on the protocol phase.

**Delsys Trigno System.** Six Avanti sensors transmit EMG signals wirelessly to the Trigno Research+ Base Station over a proprietary 2.4 GHz link. The base station sends the EMG channels to the workstation via USB, where they are ingested by QTM Software as analog input channels. This routing eliminates the need for a separate Trigno SDK process and consolidates all data acquisition within QTM.

**Qualisys Motion Capture System.** The cameras capture reflective-marker trajectories at 300 Hz and stream three-dimensional kinematic data to QTM over Ethernet. To synchronize the motion-capture and EMG recordings, the Qualisys Camera Sync Unit is configured in QTM to generate hardware TTL synchronization signals. Event generation is enabled, and the TTL signal polarity is set to negative. In the present configuration, Camera Sync Unit Out 1 operates in frequency-multiplier mode with a multiplier of 1 and a 50% duty cycle, producing a 300 Hz synchronization output with a 3333 μs period and a 1667 μs pulse duration. The output is connected to the trigger input of the Delsys Trigno Research+ Base Station using a BNC cable. QTM capture start and stop are controlled through the software-trigger function, with a 20,000 μs delay from the trigger signal to capture start or stop, a 100 ms hold-off time, and a minimum interval of 1 s between start and stop events. The Delsys base station transmits the synchronized EMG channels to the workstation through a USB connection, allowing the EMG and motion-capture data to be recorded using a common hardware timing reference.

**Workstation.** A high-performance desktop hosts two software services:

• *QTM* Software receives the EMG and motion-capture streams, synchronizes them, and exposes the combined real-time data stream to network clients through the QTM SDK.
• *MVIC Server* is a lightweight local server process (Node.js/Express) that stores per-participant MVIC normalization values and exposes them via a REST API, which the Unity application retrieves over Wi-Fi at session start.

**Meta Quest 3 (HMD).** The headset runs the Unity application as a standalone Android build. The Unity app receives synchronized EMG and kinematic data from QTM, retrieves MVIC normalization values from the MVIC Server, computes %MVIC, and renders the current visualization mode according to the participant’s randomized visualization-order sequence: avatar muscle coloring, dynamic bar indicators, or live EMG waveform graphs. Feedback is displayed either in a fully immersive VR environment or overlaid on the physical environment in MR passthrough mode.

Workstation Setup

Before each participant session, the workstation software is started in the following order: QTM Software, MVIC Server, and the Unity application on the Meta Quest 3 headset.

In QTM, the laboratory project template is loaded, the camera calibration is verified, and the Delsys EMG channels are checked as analog input channels. A short test recording is performed before the session to confirm that EMG and motion-capture data share the same start reference.

The MVIC Server is launched on the workstation as a local Node.js/Express service, and the participant-specific MVIC values are checked before running the exercise trials. The Unity application is then launched on the Meta Quest 3 headset and connected to the QTM real-time stream and MVIC Server. Before data collection, the researcher verifies that the avatar motion updates from the motion-capture stream and that the EMG visualization responds to brief voluntary deltoid contractions.

### Experimental protocol and procedures

Each participant will complete a single laboratory visit lasting approximately 120 min. The session will proceed through six sequential phases summarized in Fig. 7.

#### Phase 1: introduction, consent, and pre-screening

Upon arrival at the laboratory, participants will be briefed on the study objectives, procedures, potential risks, and their rights as research participants. Written informed consent will be obtained prior to any study procedures. Eligibility will be confirmed via a pre-screening questionnaire covering age, medical history, prior VR/MR experience, and exercise capability. The Shoulder Pain and Disability Index (SPADI) [15] will be administered as a screening instrument to confirm the absence of clinically relevant shoulder pain or disability prior to participation.

#### Phase 2: instrumentation and preparation

Phase 2 includes EMG sensor placement, reflective marker attachment, and headset fitting. For participants assigned to the VR or MR groups, the Meta Quest 3 headset will be fitted and adjusted to ensure optical clarity, comfort, and secure positioning before calibration.

##### EMG electrode placement

Six Delsys Trigno Avanti sensors will be placed bilaterally on the anterior, middle, and posterior deltoid muscles of each participant, following Surface Electromyography for the Non-Invasive Assessment of Muscles (SENIAM) recommendations [16]. Fig. 3 illustrates the deltoid placement regions, and Table 1 summarizes the placement location, orientation, starting posture, electrode size, inter-electrode distance, and reference information for each deltoid head.

**Table 1:** EMG sensor placement locations.

lectrode Location (SENIAM)	Orientation	Starting Posture	Electrode Size	IED
**· Muscle:** Anterior Deltoid **· Origin:** Anterior border, superior surface, lateral 1/3 of clavicle **· Function:** Shoulder flexion & medial rotation				
One finger width distal and anterior to the acromion	In the direction of the line between the acromion and the thumb	Sitting with the arms hanging vertically and the palm pointing inwards	Max 10 mm	20 mm
**· Muscle:** Middle (Medius) Deltoid **· Origin:** Lateral margin and superior surface of the acromion **· Function:** Shoulder abduction				
From the acromion to the lateral epicondyle of the elbow — at the greatest bulge of the muscle	In the direction of the line between the acromion and the hand	Sitting with the position of the trunk stable in relation to the arm. Stabilize the scapula if the fixation muscles are weak.	Max 10 mm	20 mm
**· Muscle:** Posterior Deltoid **· Origin:** Inferior lip of posterior border of spine of scapula **· Function:** Shoulder extension & lateral rotation				
Center the electrodes in the area about two finger-breadths behind the angle of the acromion	In the direction of the line between the acromion and the little finger	Erect sitting with the arms hanging vertically and the palm of the hand pointing inwards	Max 10 mm	20 mm

Before placement, the skin will be prepared by shaving excess hair if present, lightly abrading the site with fine sandpaper, and wiping with 70% isopropyl alcohol. Each sensor will be secured using a Delsys adhesive interface and circumferential medical tape, with the LED/logo oriented proximally to standardize alignment across participants.

##### EMG signal-quality assurance and artifact handling

EMG signal quality will be checked before and during each recording session. After sensor placement, each channel will be inspected at rest and during brief voluntary deltoid contractions to confirm clear physiological response, low resting noise, and absence of saturation, flatline behavior, or unstable wireless transmission. If a channel is noisy or unstable, the skin will be re-prepared, and the sensor will be re-secured or repositioned before recording.

During exercise trials, the researcher will monitor for signal dropout, saturation, sudden high-amplitude artifacts, or sensor movement. Trials affected by clear technical failure may be repeated or excluded, and the reason will be documented in the session log. During offline processing, raw EMG, filtered EMG, RMS envelopes, and %MVIC traces will be reviewed. Segments with severe dropout, saturation, or non-physiological artifacts will be flagged, and affected trials or channels will be excluded from the corresponding analysis when reliable feature extraction is not possible.

##### Reflective marker placement

Retroreflective markers will be attached to anatomical landmarks on the arms, shoulders, torso, pelvis, and lower limbs using the Qualisys Sports Marker Set. The marker set was selected because it supports biomechanically defined segment tracking and real-time skeleton generation through the Qualisys Skeleton Solver. The generated skeleton will be used to drive the full-body avatar in the immersive feedback application. The “Movable” markers listed in Table 2 indicate segment-tracking markers placed on body segments, such as the sternum, thigh, shin, upper arm, or pelvis, to improve segment tracking during dynamic movement. Joint centers will be estimated using functional calibration and landmark-based regression according to the Qualisys processing workflow.

**Table 2:** Detailed marker placement.

	#	Display Name	Location	Movable
	1	HeadFront	Forehead, above the nose.	-
	2	HeadL, HeadR	Just above the ear center.	-
	3	LShoulderTop, RShoulderTop	On top of the shoulder (bony prominence).	-
	4	LElbowOut, RElbowOut	On the outside of the elbow (bony prominence).	-
	5	LElbowIn, RElbowIn	On the inside of the elbow (bony prominence).	-
	6	LWristIn, RWristIn	On the inside of the wrist (bony prominence on the thumb side).	-
	7	LWristOut, RWristOut	On the outside of the wrist (bony prominence on the pinky side).	-
	8	Chest	Upper part of the sternum.	On the sternum
	9	WaistLFront, WaistRFront	On the front of the pelvis (bony prominence).	-
	10	LThighFrontLow, RThighFrontLow	Above the kneecap.	On the thigh
	11	LKneeOut, RKneeOut	On the outside of the knee (bony prominence).	-
	12	LKneeIn, RKneeIn	On the inside of the knee (bony prominence).	-
	13	LShinFrontHigh, RShinFrontHigh	Front of the shin.	On the shin
	14	LAnkleOut, RAnkleOut	On the outside of the ankle.	-
	15	LForefoot5, RForefoot5	On the base of the fifth toe.	-
	16	LForefoot2, RForefoot2	On the base of the second toe.	-
	17	SpineThoracic2	On the 2nd prominence below the biggest prominence on the top of the spine.	-
	18	LArm, RArm	On the back of the upper arm.	On the upper arm
	19	LHand2, RHand2	On the back of the hand at the base of the index finger.	-
	20	SpineThoracic12	A few cm below the midpoint of the lower tip of the shoulder blades.	-
	21	WaistBack	On the midpoint between the two prominences on the back of the pelvis.	-
	22	WaistL, WaistR	On the sides of the pelvis (bony prominence).	On the pelvis
	23	LHeelBack, RHeelBack	Back of the heel.	-

#### Phase 3: baseline and calibration measurements

Phase 3 comprises three sequential activities: (1) MVIC testing to establish EMG normalization references, (2) exercise familiarization using instructional videos, and (3) baseline exercise trials without immersive feedback.

##### MVIC protocol

**Fig. 4:**
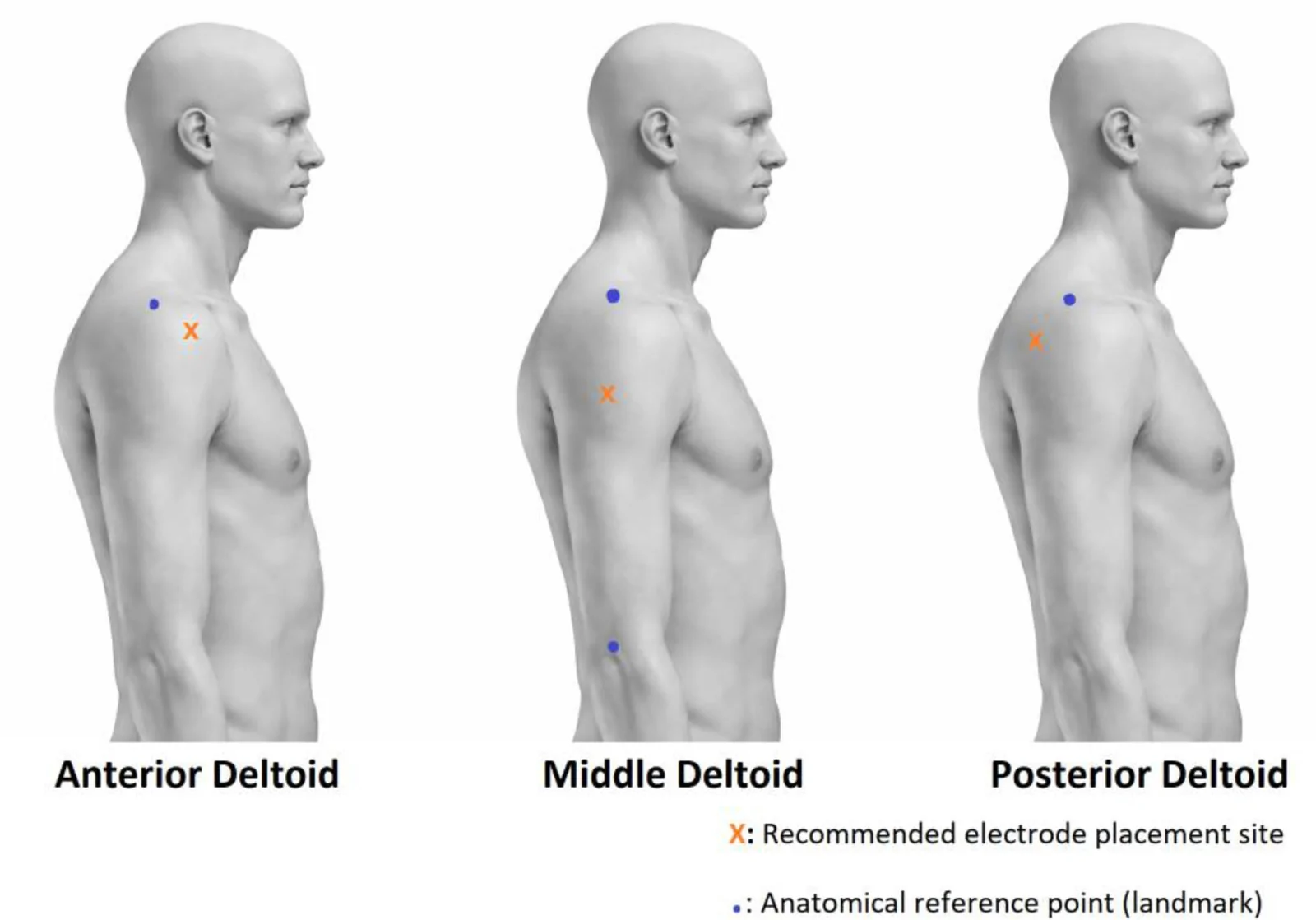
MVIC using a resistance band (A: anterior, B: middle, C: posterior).

MVIC testing will be used to establish per-muscle normalization references for all subsequent %MVIC calculations [16,17]. Contractions will be performed bilaterally, with the dominant side tested first. Resistance bands will be used to provide external resistance, as shown in Fig. 4, and the same research assistant will deliver standardized verbal encouragement throughout each trial (“Push! Push! Keep going!”).

**Positioning by muscle:**

**Anterior deltoid:** The participant sits with feet flat and trunk supported against the chair back. The arm is positioned at 90° shoulder flexion with the elbow extended and forearm neutral. The resistance band is secured at the hand/wrist and anchored to resist further shoulder flexion, and the participant pushes maximally upward.

**Middle deltoid:** The participant sits with the trunk stable. The arm is positioned at 90° abduction in the coronal plane with the elbow extended. The resistance band is secured at the hand/wrist and anchored to resist further abduction, and the participant pushes maximally outward.

**Posterior deltoid:** The participant sits with the trunk inclined forward. The arm is positioned at approximately 90° shoulder abduction/horizontal abduction with the elbow extended. The resistance band is secured at the hand/wrist and anchored to resist further horizontal abduction, and the participant pushes maximally backward.

Each muscle will be tested for 3 trials per side, resulting in 18 MVIC trials per participant. Each trial will include a 5 s maximal contraction, with 60 s rest between trials. The testing order will be fixed as anterior deltoid, middle deltoid, and posterior deltoid. The peak RMS value from the central 5 s of the highest-amplitude trial will be retained as the MVIC reference for the corresponding muscle and side. Exercise EMG will be expressed as:

**%MVIC = (RMS_exercise / RMS_MVIC_peak) × 100**

##### Exercise familiarization and baseline trials

**Fig. 5:**
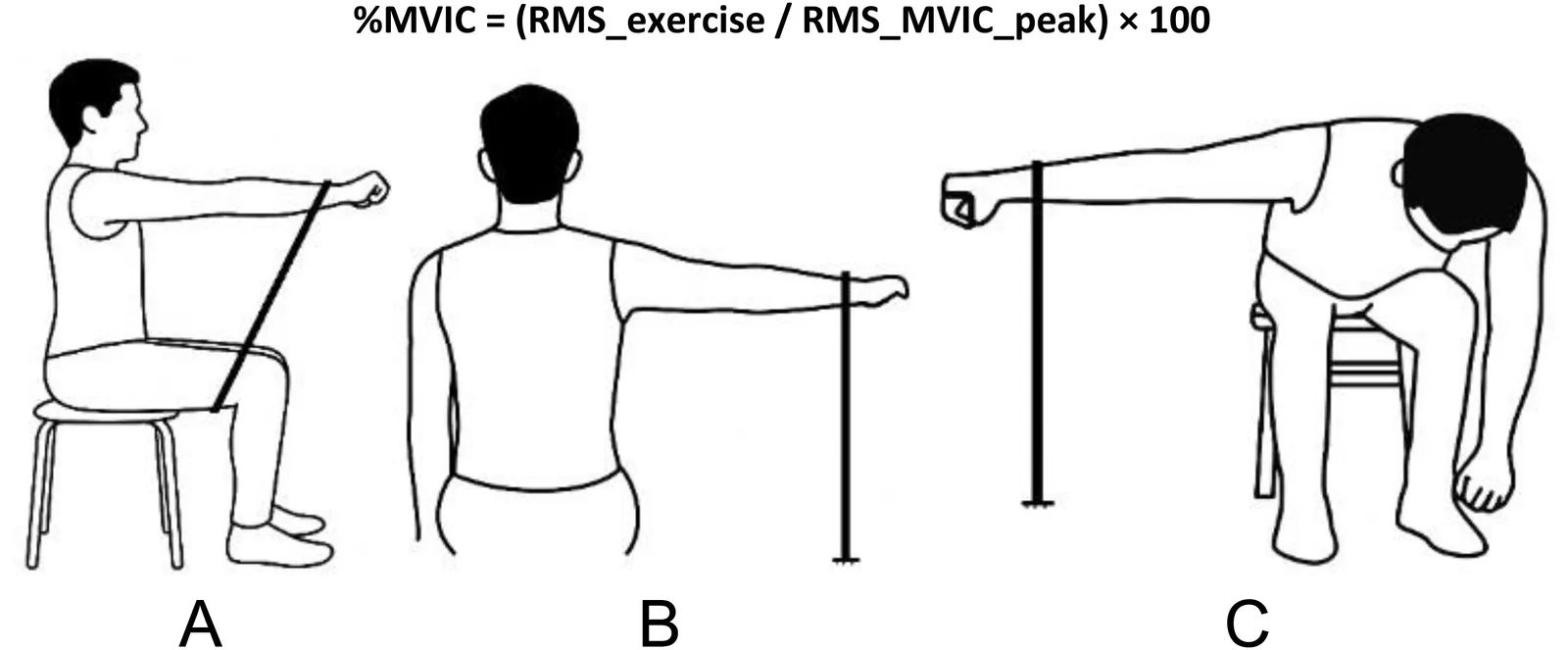
Experiment exercises.

Following MVIC testing, participants will view a short instructional video for each standardized shoulder exercise to ensure consistent understanding of the target technique and prescribed movement range. The five exercises illustrated in Fig. 5 will be performed in the following fixed order:

1. Alternating Dumbbell Front Raise
2. Standing Dumbbell Shoulder Press
3. Dumbbell Lateral Raise
4. Bent Arm Lateral Raise
5. Reverse Dumbbell Fly

Participants will then perform five repetitions of each exercise without immersive feedback, with all EMG sensors and motion-capture markers active. These baseline trials will serve as the within-participant reference for subsequent performance comparisons. The working dumbbell load will be selected as the load with which the participant can complete all repetitions using acceptable technique, as judged by a trained research assistant using a standardized technique checklist. This load will be recorded and used for the corresponding experimental trials in Phase 4.

#### Phase 4: experimental exercise trials

Participants will perform the five standardized shoulder exercises in the fixed order established during familiarization for all three visualization modes. Each exercise will include five repetitions using the individually determined working load recorded during Phase 3. Rest intervals of 2–3 min will be provided between exercises to minimize fatigue, and participants may request additional rest if needed. A trial will be stopped if the participant reports pain, dizziness, excessive fatigue, simulator sickness symptoms, skin irritation, or any discomfort that raises safety concerns. Any stopped, repeated, or excluded trial will be documented in the session log.

**Fig. 6:**
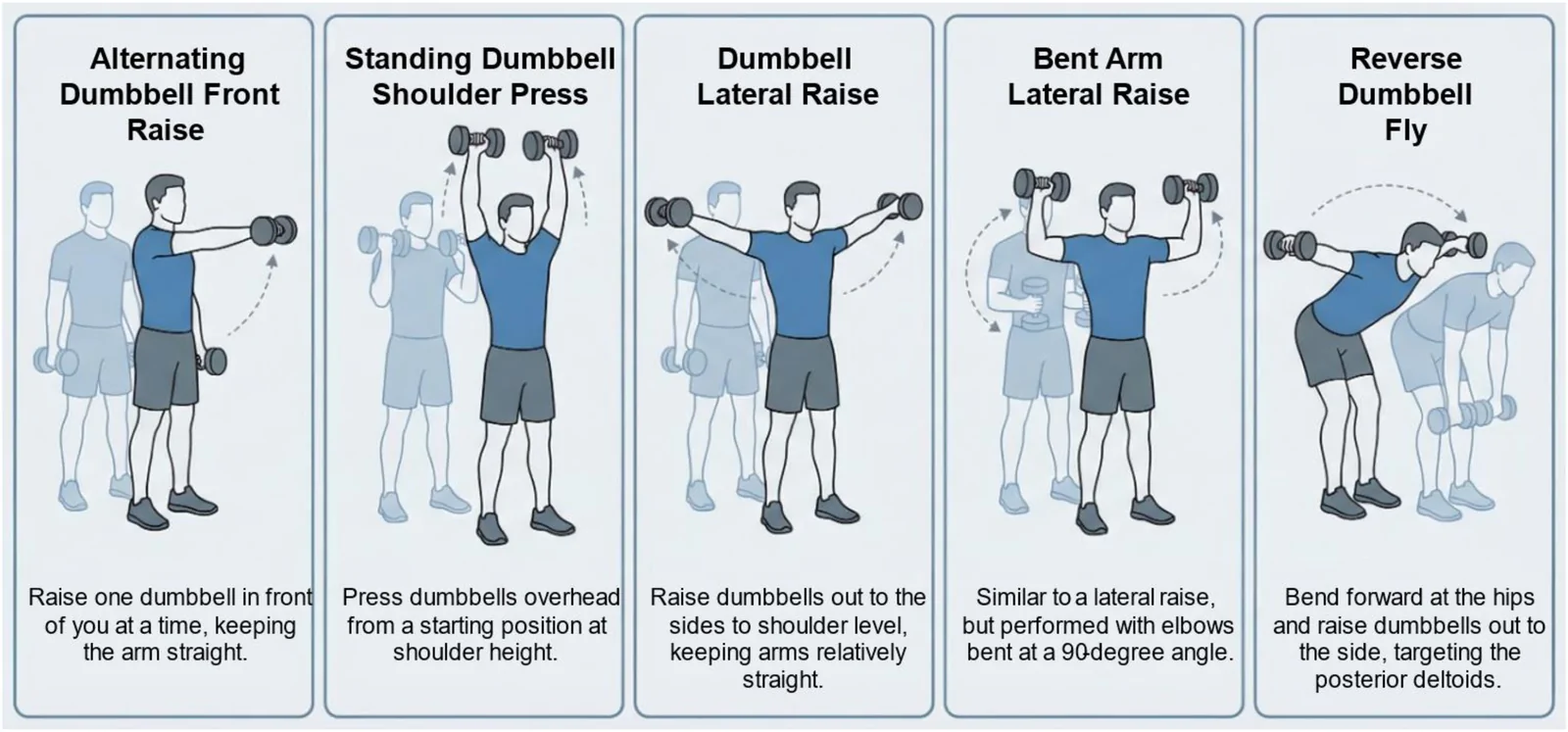
Avatar visualization with muscle-activity color coding.

**VR and MR Groups:** Participants will wear the Meta Quest 3 headset and receive real-time EMG biofeedback through all three visualization modes in a randomized order:

• **Avatar Mode:** A 3D avatar mirrors the participant’s movements, as shown in Fig. 6, with deltoid regions colored according to EMG amplitude.

**Fig. 7:**
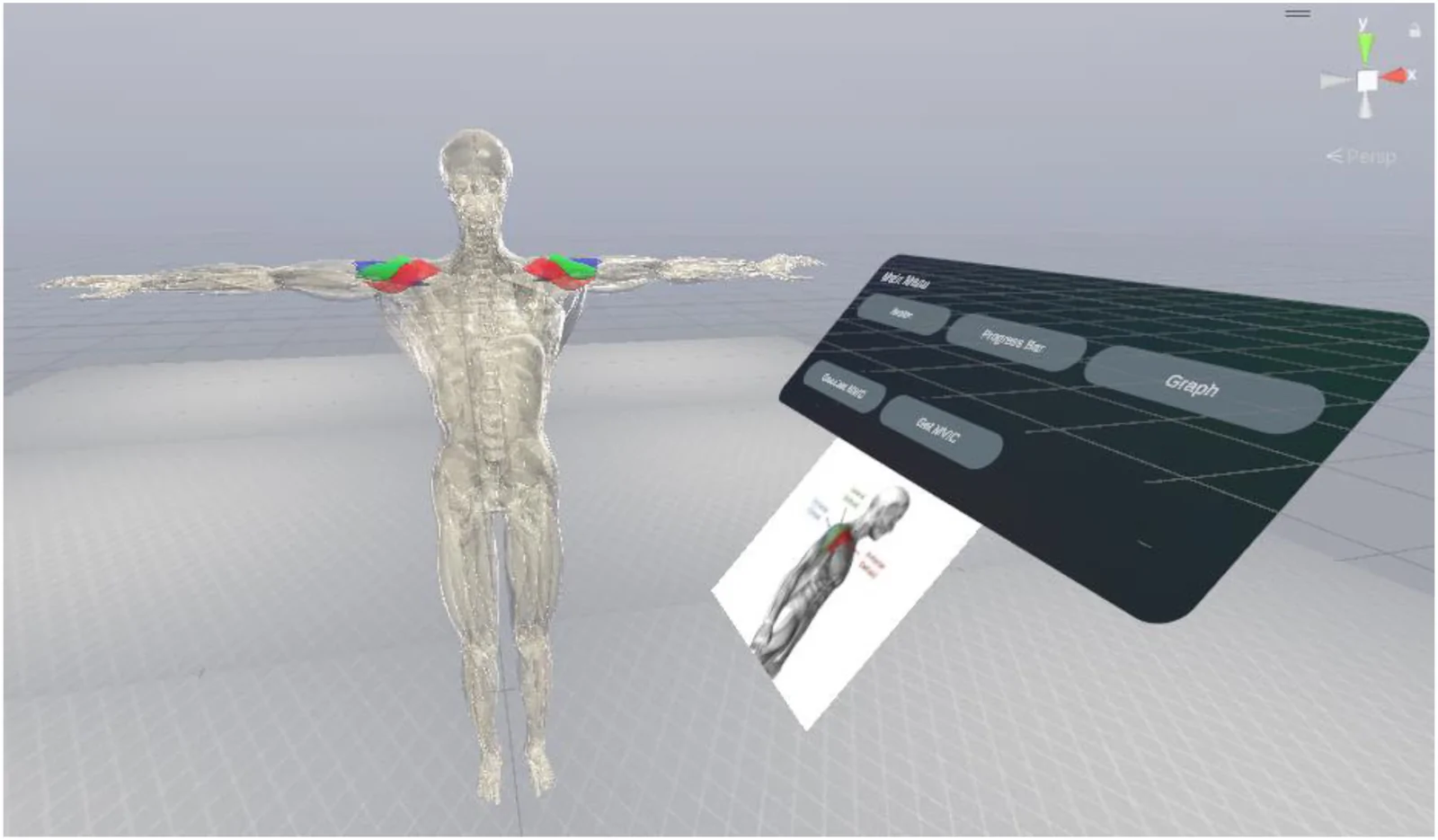
Experimental protocol and procedures.
• **Bar Mode:** Dynamic bar indicators show EMG amplitude for each monitored muscle.
• **Graph Mode:** Scrolling waveform graphs display filtered EMG signals in real time.

In the VR condition, feedback will be displayed in a fully immersive virtual environment. In the MR condition, feedback will be overlaid onto the participant’s view of the physical laboratory using the headset’s passthrough cameras.

**Control Group:** Participants will perform the same exercises without augmented feedback and as 3 blocks matching the VR and MR group number of repetitions and rest periods, while wearing the same EMG sensors and motion-capture markers to maintain equivalent instrumentation burden.

During rest intervals, participants will complete brief in-session questionnaires, including the Borg Rating of Perceived Exertion (RPE) scale [18] to assess perceived effort for each exercise.

#### Phase 5: post-exercise assessments (VR and MR groups only)

After completing all exercise trials, participants in the VR and MR groups will complete two post-exercise questionnaires:

• **System Usability Scale (SUS)** [19]**:** a 10-item questionnaire assessing perceived usability of the immersive biofeedback system.
• **Simulator Sickness Questionnaire (SSQ)** [20]**:** a 16-item questionnaire assessing symptoms of simulator sickness, including nausea, oculomotor discomfort, and disorientation.

The RPE assessment will not be repeated in this phase because it is collected during the rest intervals in Phase 4 for all groups.

#### Phase 6: debriefing and equipment removal

All sensors, markers, and headset equipment will be carefully removed. Participants will be debriefed, given an opportunity to ask questions, and monitored for any immediate discomfort. Any adverse events, including muscle soreness, skin irritation, or simulator-sickness symptoms, will be documented.

### Outcome measures

#### Primary outcomes: objective measures

**Joint Angle Accuracy:** Deviation of measured shoulder joint angles, including flexion, abduction, and rotation, from the prescribed target range for each exercise. Lower deviation indicates greater movement accuracy.

**Muscle Activation Patterns:** EMG amplitude normalized to MVIC for each monitored deltoid muscle. Metrics include mean activation, peak activation, and activation timing.

**Movement Metrics:**

• **Range of Motion (ROM):** Maximum angular displacement achieved during each repetition.
• **Movement Speed:** Peak and mean angular velocity during concentric and eccentric phases.
• **Movement Smoothness:** Smoothness of the velocity profile quantified using spectral arc length (SPARC) and movement-unit count.
• **Compensatory Movements:** Trunk motion or contralateral limb movement during unilateral exercises, used to identify compensation strategies.

**Task Completion Time:** Total time required to complete five repetitions of each exercise.

#### Secondary outcomes: subjective measures

**Perceived Exertion:** RPE score recorded after each exercise.

**Simulator Sickness:** SSQ total and subscale scores for nausea, oculomotor discomfort, and disorientation in the VR and MR groups.

**System Usability:** SUS score from 0 to 100 in the VR and MR groups, with scores above 68 indicating above-average usability [19].

#### Planned statistical analysis

Descriptive statistics will be used to summarize all objective and subjective outcomes. Continuous variables will be reported as mean ± standard deviation or median and interquartile range, depending on data distribution. For outcomes measured repeatedly across exercises and visualization modes, linear mixed-effects models will be used where appropriate, with participant included as a random effect and group, exercise, and visualization mode included as fixed effects. Model assumptions will be checked before analysis, and non-parametric alternatives will be used when required. Statistical significance will be set at (p < 0.05), with effect sizes and 95% confidence intervals reported where appropriate.

### Data processing

Data processing occurs in two stages: a **runtime** pipeline that drives real-time XR biofeedback during the experimental session, and a **post-session** pipeline that processes recorded data offline to compute objective outcome measures.

#### Runtime processing pipeline

The runtime pipeline is executed by the Unity application running as a standalone build on the Meta Quest 3 headset. Its purpose is to generate low-latency normalized EMG values for real-time visual feedback. Processing occurs across three stages.

##### Stage A — sensor hardware (Delsys Trigno Avanti)

Each Trigno Avanti sensor performs onboard EMG conditioning using a 20–450 Hz hardware band-pass filter before wireless transmission to the Trigno Research+ Base Station (SP-W02). The base station sends digitized EMG channels to the workstation, where they are ingested by QTM Software.

##### Stage B — QTM software (workstation)

QTM Software receives the EMG channels and 3D marker trajectories, synchronizes both streams using the shared TTL trigger, and packages them into real-time frames for streaming to Unity through the QTM SDK.

##### Stage C — Unity application (Meta Quest 3)

The Unity application polls QTM for EMG data over Wi-Fi. For each EMG channel, Unity computes RMS amplitude from the latest QTM analog sample packet, applies a 20-update rolling RMS window, and normalizes the resulting value by the corresponding MVIC reference to produce a %MVIC value. The six %MVIC values are then sent to the visualization controller: avatar muscle coloring, dynamic bar indicators, or live waveform graphs. The feedback is rendered in either VR or MR mode according to group allocation.

#### Post-session processing pipeline

Following each session, raw QTM exports containing 3D marker trajectories and analog EMG channels are processed offline in Python (v3.11) using SciPy and NumPy to compute the objective outcome measures. Questionnaire outcomes are scored separately according to their standard scoring procedures. The offline pipeline applies higher-precision processing than the runtime pipeline because it is not constrained by real-time latency.

##### EMG signal processing

The following steps are applied to each EMG channel for every trial:

1. **DC offset removal:** mean subtraction per channel and trial.
2. **Band-pass filtering:** 4th-order zero-phase Butterworth filter, 20–450 Hz.
3. **Notch filtering:** 2nd-order IIR notch filter at 50 Hz to reduce power-line interference.
4. **Full-wave rectification:** absolute value of the filtered signal.
5. **RMS envelope extraction:** the RMS envelope is computed using a sliding window as:
  $$\text{RMS}\left[n\right]=\sqrt{\frac{1}{N}\sum_{i=0}^{N-1}x^{2}\left[n-i\right]}$$

where n is the current sample index, i is the sample offset within the sliding window, x is the filtered EMG signal, and N is the number of samples in the RMS window. A 50 ms RMS window is used for exercise trials for smoother visualization.

**6. MVIC normalization:** exercise EMG is expressed as a percentage of maximum voluntary isometric contraction (%MVIC) using the corresponding per-muscle, per-side MVIC reference:

$\%\text{MVIC}\left[n\right]=\frac{\text{RM}S_{\text{exercise}}\left[n\right]}{\text{RM}S_{\text{MVIC},\text{peak}}}\times \;100$where n is the current sample index, $\text{RM}S_{\text{exercise}}\left[n\right]\;$is the exercise RMS value at sample n, and $\text{RM}S_{\text{MVIC},\text{peak}}$ is the peak RMS value obtained from the central 5 s of the highest-amplitude MVIC trial.

**7. Feature extraction:** mean %MVIC, peak %MVIC, time-to-peak activation, and activation duration using a 10% MVIC onset/offset threshold.

##### Kinematic processing

Raw 3D marker trajectories are processed using the Qualisys Sports Marker Set segment definitions. The pipeline computes shoulder and elbow joint angles, joint-angle accuracy, range of motion, angular velocity, movement smoothness, and compensatory motion. Compensatory movement is quantified using trunk displacement and contralateral limb excursion during unilateral exercises.

#### Temporal alignment and repetition segmentation

EMG and kinematic data are aligned using the common hardware timing reference established by the TTL synchronization signal. Individual repetitions are segmented using kinematic event detection based on local minima and maxima of the primary joint-angle trajectory, identified from zero-crossings of the smoothed angular velocity signal.

#### Real-time software latency assessment

Unity-side software latency was measured using a monotonic timestamp logger integrated into the application. For each QTM packet processed by Unity, the logger recorded the Unity-side packet reception time, EMG processing start and end times, and the time at which the corresponding visualization value was applied. Processing latency was calculated as the interval between processing start and end. Receive-to-visualization latency was calculated as the interval between Unity-side QTM packet reception and visualization update.

Across complete six-channel update cycles, receive-to-visualization latency had a median of 11.0 ms, a mean of 11.1 ± 5.1 ms, a 95th percentile of 18.2 ms, and a maximum of 35.3 ms. Unity-side EMG processing had a median latency of 0.0035 ms, a 95th percentile of 0.0050 ms, and a maximum of 0.3045 ms.

These results represent latency within the Unity software pipeline after each QTM packet was received.

**Protocol validation**

The protocol was validated using an end-to-end pilot dataset from one participant (MR_003). This pilot validation was performed as an engineering and workflow check before full data collection. Validation focused on confirming that the acquisition, synchronization, segmentation, and post-session analysis workflow could generate interpretable kinematic and EMG outputs across the three immersive feedback visualization modes: avatar, bar, and graph. This validation was not intended to evaluate clinical or performance efficacy, but to verify whether the protocol can produce analyzable multimodal outputs under the planned experimental conditions.

**Fig. 8:**
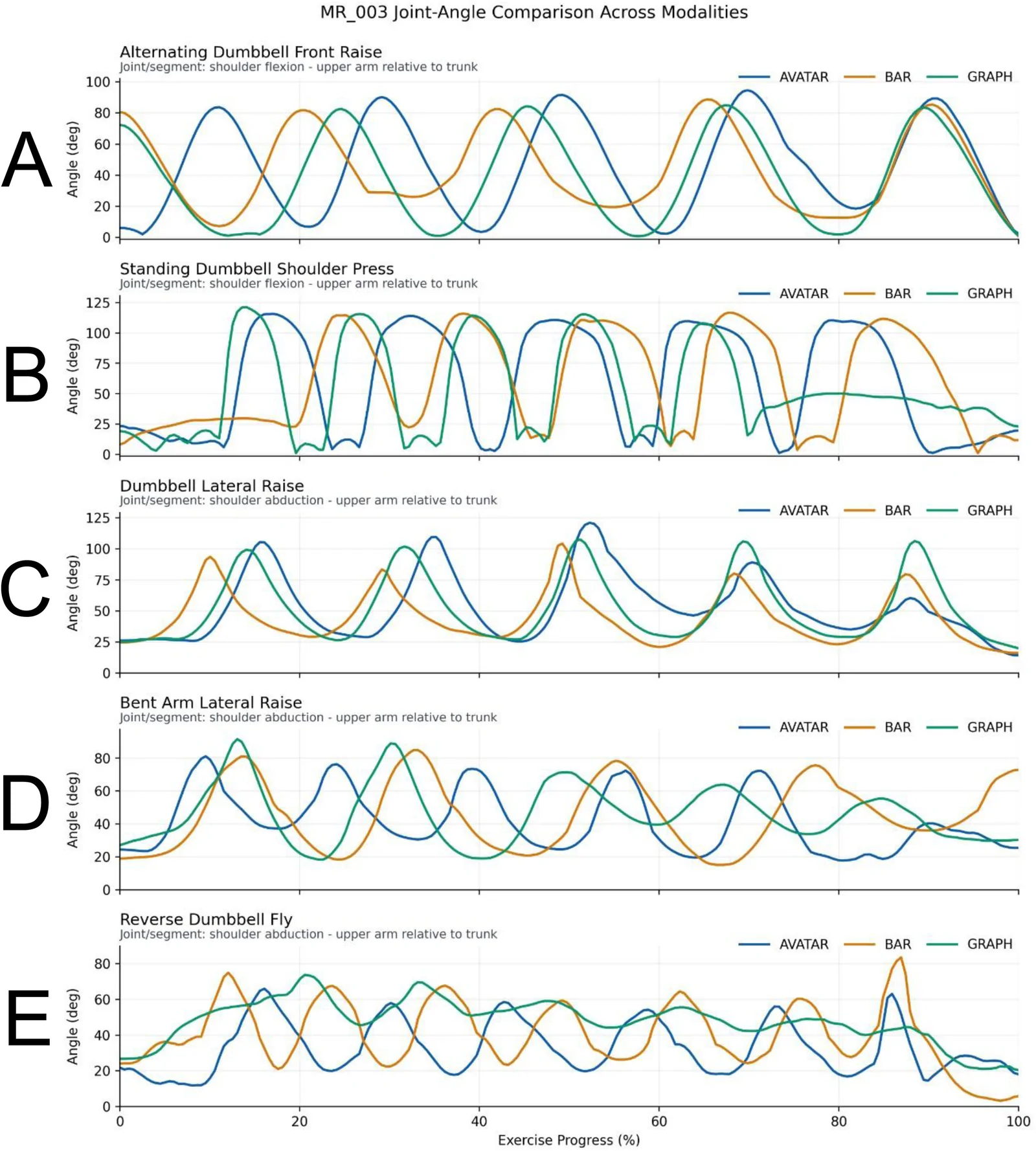
Joint-angle trajectories from pilot participant MR_003 across avatar, bar, and graph visualization modes. The horizontal axis represents normalized exercise progress from 0% to 100%, and the vertical axis represents the primary shoulder angle in degrees. The blue, orange, and green traces correspond to the avatar, bar, and graph modes, respectively. Panel A shows shoulder flexion during the alternating dumbbell front raise; Panel B shows shoulder flexion during the standing dumbbell shoulder press; Panel C shows shoulder abduction during the dumbbell lateral raise; Panel D shows shoulder abduction during the bent-arm lateral raise; and Panel E shows shoulder abduction during the reverse dumbbell fly. Repeated peaks and troughs represent successive exercise repetitions and demonstrate that the motion-capture pipeline can preserve exercise-specific joint-angle patterns across visualization modes.

The joint-angle trajectories showed that the five standardized shoulder exercises could be segmented and visualized across all three feedback modes (Fig. 8). Exercises 1 and 2 captured shoulder flexion relative to the trunk, while Exercises 3–5 captured shoulder abduction relative to the trunk. Across conditions, the trajectories showed repeated movement cycles consistent with the prescribed five-repetition structure, indicating that the kinematic pipeline can capture exercise-specific movement patterns and compare temporal execution profiles between visualization modes.

**Fig. 9:**
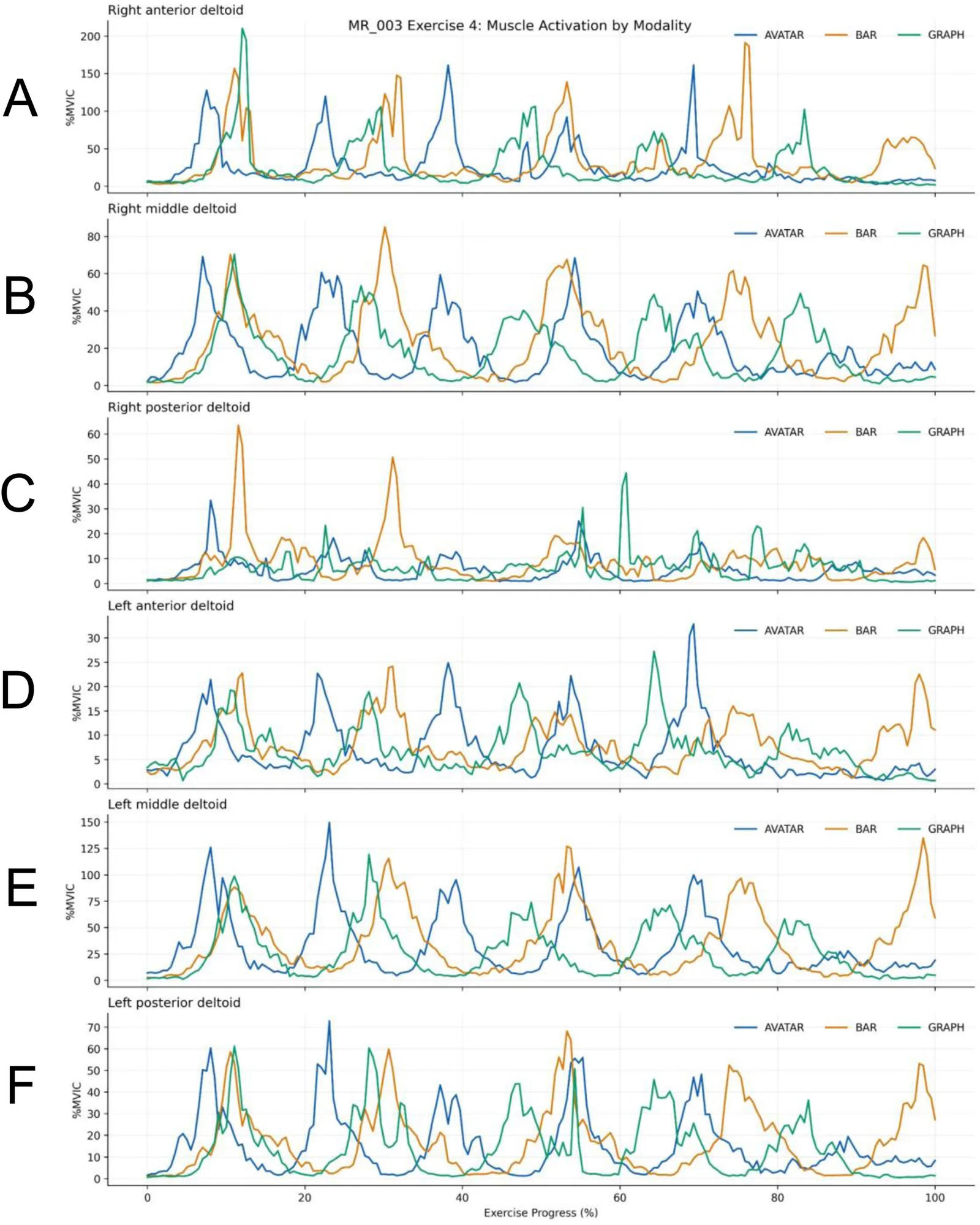
Normalized deltoid muscle activation from pilot participant MR_003 during Exercise 4 across avatar, bar, and graph visualization modes. The horizontal axis represents normalized exercise progress from 0% to 100%, and the vertical axis represents muscle activation normalized to maximum voluntary isometric contraction (%MVIC). The blue, orange, and green traces correspond to the avatar, bar, and graph modes, respectively. Panels A–C show the right anterior, middle, and posterior deltoid, while Panels D–F show the corresponding left-side muscles. The traces illustrate time-varying, channel-specific muscle recruitment throughout the exercise.

The EMG validation output for Exercise 4 showed that the six monitored deltoid channels could be processed and normalized to a percentage of maximum voluntary isometric contraction (%MVIC) across avatar, bar, and graph modes (Fig. 9). The activation profiles demonstrated time-varying muscle recruitment patterns across the right and left anterior, middle, and posterior deltoid channels. The presence of distinct activation peaks across channels and modes indicates that the EMG workflow can preserve channel-specific information and generate interpretable normalized activation traces for subsequent feature extraction.

**Fig. 10:**
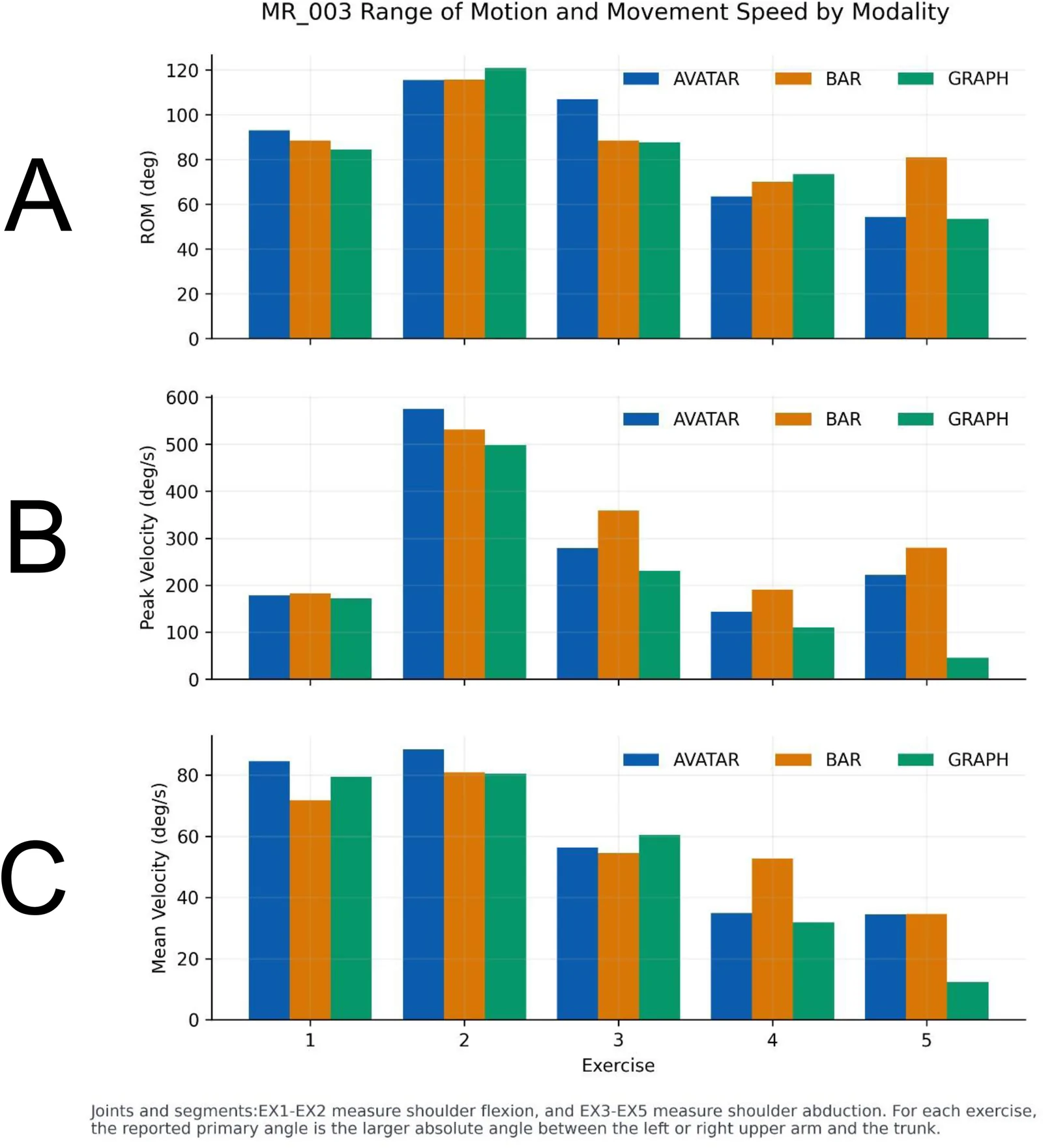
Range of motion and movement-speed metrics from pilot participant MR_003 across avatar, bar, and graph visualization modes. The blue, orange, and green bars represent the avatar, bar, and graph modes, respectively. Panel A presents range of motion in degrees, Panel B presents peak angular velocity in degrees per second, and Panel C presents mean angular velocity in degrees per second for Exercises 1–5. Exercises 1 and 2 are based on shoulder flexion, whereas Exercises 3–5 are based on shoulder abduction. For each exercise, the reported primary angle is the larger absolute angle between the left or right upper arm and the trunk.

Summary kinematic metrics further supported the feasibility of the analysis pipeline (Fig. 10). Range of motion, peak angular velocity, and mean angular velocity were successfully extracted for each of the five exercises and compared across the three feedback modes. The bar plots show that the workflow can reduce continuous motion-capture data into exercise-level metrics while preserving differences in movement amplitude and speed across visualization modes. Together, these pilot outputs support the feasibility of the proposed protocol for collecting synchronized EMG and motion-capture data, segmenting standardized shoulder exercises, and generating objective outcome measures for the full experimental dataset.

Because validation was performed using a single participant, the results should be interpreted only as a workflow and feasibility check. Group-level comparisons, statistical testing, and conclusions regarding the effect of VR and MR delivery or visualization mode will require the complete participant sample.

## Limitations

• The single-session design precludes assessment of motor learning effects, skill retention, and long-term adherence outcomes that are critical for evaluating the clinical utility of immersive rehabilitation systems.
• The exclusive focus on healthy young adults may limit the generalizability of findings to clinical populations with shoulder pathology, older adults, or individuals with physical or cognitive impairments relevant to rehabilitation.
• Because participants in the VR and MR groups experience all three visualization modes in a single session, learning, fatigue, or carryover effects between visualization blocks may influence performance. Randomized visualization order and rest intervals are used to reduce these effects, but they cannot be fully eliminated.

## CRediT author statement

Mazen Talaat Haggag, Mohammed Ibrahim Awad, Ahmed Mohamed Elbokl, and Bassem Amin Abdullah participated in the design of the protocol. Mazen Talaat Haggag wrote the protocol manuscript. Mazen Talaat Haggag, Mohammed Ibrahim Awad, Ahmed Mohamed Elbokl, and Bassem Amin Abdullah reviewed and agreed to the final version of the protocol.

## Supplementary material and/or additional information

The source code and implementation materials supporting this protocol are available at:

Unity XR application: https://github.com/MazenTalaat/HCM_EMG_XR

MVIC Server: https://github.com/MazenTalaat/HCM_EMG_XR/tree/main/MVIC_Server

## Declaration of interests

The authors declare that they have no known competing financial interests or personal relationships that could have appeared to influence the work reported in this paper.

## Data Availability

The data that has been used is confidential.
